# Study on multi-modal feature fusion machine learning classification model of Chinese and Western medicine for depressive symptoms in middle-aged and older patients with chronic low back pain

**DOI:** 10.3389/fpsyt.2026.1761254

**Published:** 2026-08-13

**Authors:** Bo Jin, Juan Gao, Chenying He, Xiaohong Lei

**Affiliations:** 1 Department of Emergency, The Second Affiliated Hospital of Hunan University of Traditional Chinese Medicine, Changsha, Hunan, China; 2 Department of Nursing, The Second Affiliated Hospital of Hunan University of Traditional Chinese Medicine, Changsha, Hunan, China; 3 Department of Orthopedics, The Second Affiliated Hospital of Hunan University of Traditional Chinese Medicine, Changsha, Hunan, China

**Keywords:** chronic low back pain, classification model, depression, machine learning, middle-aged and older people, traditional Chinese medicine syndrome

## Abstract

**Objective:**

To construct a machine learning classification model integrating multi-modal features of Chinese and Western medicine for screening the risk of depressive symptoms in middle-aged and older patients with chronic low back pain (CLBP).

**Methods:**

A cross-sectional design included 370 CLBP patients aged ≥45 years admitted to our hospital from January 2022 to June 2024. Based on PHQ-9 scores, they were classified into depression (≥10) and non-depression (<10) groups. Chinese and Western medicine characteristics were collected and randomly split into training and validation sets (7:3 ratio). In the training set, Least Absolute Shrinkage and Selection Operator(LASSO) and multivariate logistic regression identified risk factors for depressive symptoms. Gradient boosting machine (GBM) and random forest (RF) models were then built using these factors. Model performance was assessed via AUC, sensitivity, specificity, and calibration curves, with DeLong tests comparing models. Additionally, a temporal validation cohort of 156 patients admitted from July 2024 to June 2025 was used to evaluate model stability over time.

**Results:**

The prevalence of depressive symptoms in the training set was 28.20%. Multivariate analysis identified VAS score, ODI score, PSQI score, qi deficiency constitution, and blood stasis constitution as independent risk factors for depressive symptoms, while sleep duration served as a protective factor (all P<0.05). The GBM model achieved AUCs of 0.927 and 0.837 in the training and validation sets, respectively. The RF model achieved AUCs of 0.998 and 0.811 in the training and validation sets, respectively. Both models demonstrated good calibration in both sets. The DeLong test indicated no statistically significant difference in performance between the two models in the training or validation sets (P>0.05). In temporal validation, the AUCs for the GBM and RF models were 0.956 and 0.952, respectively, with no statistically significant difference between the two models (P = 0.751).

**Conclusion:**

Both GBM and RF models incorporating multimodal features from traditional Chinese and Western medicine demonstrated promising preliminary classification performance for screening depressive symptoms in middle-aged and older patients with CLBP, suggesting their potential utility as screening tools in clinical settings. Further multicenter external validation is needed to confirm the generalizability of these findings.

## Introduction

Chronic low back pain (CLBP) in middle-aged and older individuals is defined as a pain syndrome lasting 12 weeks or longer, occurring in the region between the 12th rib and gluteal fold. It is a common condition that significantly affects quality of life in this population. Based on etiology, CLBP can be classified into primary low back pain and specific low back pain (1). Research indicates that the prevalence of CLBP in China is approximately 17.6% (2), with a marked increase associated with advancing age. Among individuals aged 40–69 years, the prevalence ranges from 28% to 42%, particularly in women aged 40–80 years (3). The Global Burden of Disease Study conducted in 2019 (4) revealed that CLBP ranks sixth among the causes of disability in individuals aged 50–74 years. In addition to directly causing functional impairment, CLBP is closely associated with an increased risk of falls (5), employment difficulties, and heightened socioeconomic burden, thereby constituting a significant public health issue.

Notably, there is a close association between CLBP and depressive symptoms. Studies have shown that approximately 30–50% of patients with chronic CLBP experience comorbid depressive symptoms (6). This relationship creates a vicious cycle characterized by “pain–functional impairment–depression,” which severely affects recovery and overall quality of life. Currently, the clinical identification of depression risk primarily relies on passive inquiry after symptom onset, and effective early warning systems are lacking. Although contemporary medical classification research has begun to incorporate psychosocial factors along with somatic pain elements, its discriminative performance remains suboptimal. Traditional Chinese medicine (TCM) offers unique advantages in understanding psychosomatic disorders. TCM theory emphasizes an integrated perspective described as “the unity of body and mind,” incorporating concepts such as “the kidney being the residence of low back health” and “the liver governing smooth flow.” It proposes pathophysiological theories, including “kidney deficiency as the root cause,” “blood stasis as the manifestation,” and “liver stagnation as transformation,” thereby providing a comprehensive theoretical framework for understanding this comorbidity from both physical and emotional perspectives (7, 8). However, the qualitative and subjective nature of TCM syndromes limits their applicability to large-scale population studies.

With advances in artificial intelligence technology, machine learning algorithms have been increasingly applied in the construction of medical classification models owing to their strong feature extraction and pattern recognition capabilities. Among these approaches, Gradient Boosting Machine (GBM) and Random Forest (RF) models are particularly effective in handling nonlinear relationships while reducing the risk of overfitting and have demonstrated strong performance in disease risk classification (9–11). To date, studies integrating TCM syndrome information with modern medical indicators using machine learning methods to identify depression risk among patients with CLBP are lacking. Therefore, this study proposes integrating multimodal features from Chinese and Western medicine and applying machine learning algorithms to construct a classification model for identifying depressive symptom risk in middle-aged and older patients with CLBP, thereby providing data-driven decision support to promote preventive interventions through integrated Chinese and Western medicine.

## Materials and methods

### Materials

A cross-sectional survey was conducted. A total of 370 middle-aged and older patients (aged ≥45 years) with CLBP who visited our hospital between January 2022 and June 2024 were enrolled as study subjects. The inclusion criteria were as follows: ① meeting the diagnostic criteria for CLBP (12), with a disease duration of at least three months; ② age ≥45 years; and ③ clear consciousness and ability to cooperate in completing the questionnaire. The exclusion criteria included: ① severe dementia, a history of psychiatric disorders or depression, or comorbid neurodegenerative diseases; ② CLBP caused by specific pathological conditions such as tumors, infections, or fractures; ③ significant cognitive impairment or communication difficulties; ④ recent use of hormones, sedatives, antipsychotics, or antidepressants; ⑤ severe cardiac, hepatic, or renal dysfunction; ⑥ serious impairments of consciousness, hearing, or speech; ⑦ presence of epilepsy, infections, malignant tumors, infectious diseases, hematological disorders, or thyroid diseases; and ⑧ pregnancy or breastfeeding. To verify the temporal stability of the model, 156 middle-aged and older patients with CLBP treated at our hospital from July 2024 to June 2025 were included as a temporal validation cohort. This study, based on a cross-sectional design, aimed to develop a machine learning classification model to identify depressive symptoms rather than to predict future onset. This study was approved by the Ethics Committee of the Second Affiliated Hospital of Hunan University of Traditional Chinese Medicine. The requirement for informed consent was waived by the ethics committee due to the retrospective nature of the study. The study was conducted in accordance with the Declaration of Helsinki.

This was a single-center study conducted at the Second Affiliated Hospital of Hunan University of Traditional Chinese Medicine, a tertiary teaching hospital in Changsha, Hunan Province, China.

## Methods

### Multimodal feature collection

Sociodemographic and Western medicine–related clinical characteristics included sex(male/female), age, duration of low back pain(>12 months/≤12 months), body mass index (BMI), education level(junior high school or below/senior high school or above), marital status (married/unmarried/separated/divorced/widowed), and the following variables classified as yes/no: diabetes, hypertension, hyperlipidemia, smoking, alcohol consumption, family history of depression. Pain visual analog scale (VAS) score, Oswestry Disability Index (ODI), sleep quality assessed using the Pittsburgh Sleep Quality Index (PSQI), and sleep duration. TCM syndromes include *qi* deficiency, blood stasis, phlegm dampness, damp heat, and other types.

### Characteristics of TCM

TCM constitution types: According to the relevant literature (13), a standardized scale was used for evaluation, including nine constitution types: *qi* deficiency, blood deficiency, and blood stasis. These were simplified and merged into six types (*qi* deficiency, blood deficiency, blood stasis, phlegm dampness, gentleness, and other constitutions). A total score ≥40 was classified as the corresponding type of constitution.

TCM syndrome differentiation: Two TCM physicians with more than five years of clinical experience independently assessed patients according to the TCM Disease Diagnosis and Curative Effect Standards, primarily including qi stagnation and blood stasis syndrome, *qi* deficiency and blood stasis syndrome, and cold-dampness obstruction syndrome. Discrepancies were resolved by consultation with a third-party expert (yes/no for each syndrome).

### Depressive symptom grouping

The Patient Health Questionnaire-9 (PHQ-9) was used to assess depressive symptoms over the previous two weeks. The PHQ-9 consists of nine items, each scored as 0 (not at all), 1 (every several days), 2 (more than half a day), or 3 (nearly every day). The total score ranged from 0 to 27, with higher scores indicating more severe depressive symptoms. A total score of ≥10 was considered the screening threshold for depression.

### Research tools and data collection

The data were collected by uniformly trained research nurses and attending TCM physicians. After training, consistency tests were performed to ensure data reliability. Standardized data collection forms were used, and the data were entered twice into Excel and cross-checked to minimize entry errors.

### Data preprocessing

Data preprocessing included the following steps: data cleaning, feature encoding, standardization, and feature selection. A total of 370 participants were randomly divided into a training set (n=266) and an internal validation set (n=104) at a 7:3 ratio. An additional 156 participants were collected as a temporal validation cohort to assess model stability. A total of 21 multi-modal features were initially included in this study, comprising 16 sociodemographic and Western medicine–related clinical features and 5 TCM constitution features. Detailed variable definitions and assignments are presented in **Table 1**. All features underwent completeness checks during data collection, with no missing values identified; therefore, no imputation was performed. Outliers in continuous variables were identified using boxplot methods combined with clinical expertise and were verified and corrected accordingly. For categorical variables, binary variables were directly assigned values of 0/1. Continuous features (e.g., age, BMI, disease duration, VAS, ODI, PSQI score, and sleep duration) were standardized using Z-score normalization prior to model training, with standardization parameters derived from the training set and subsequently applied to the internal validation and temporal validation sets. For feature selection, LASSO regression combined with multivariable logistic regression was employed in the training set to identify predictors. During model training, 10-fold cross-validation was used to optimize parameters and prevent overfitting.

**Table 1:** Demographic characteristics of middle-aged and older patients with chronic low back pain and univariate analysis of depressive symptoms.

Factors	Depression group (n=105)	Non-depression group (n = 265)	t/χ^2^	P
Sex[n(%)]			0.199	0.656
Male	50(47.62)	133(50.19)		
Female	55(52.38)	132(49.81)		
Age (years)	58.07 ± 7.57	59.09 ± 7.15	1.226	0.221
The course of low back pain [month, n (%)]			0.400	0.527
>12	41(39.05)	113(42.64)		
≤12	64(60.95)	152(57.36)		
BMI [kg/m2, n(%)]			3.048	0.384
≤18.4	12(11.43)	31(11.70)		
18.5-23.9	52(49.52)	141(53.21)		
24.0-27.9	30(28.57)	55(20.75)		
≥28	11(10.48)	38(14.34)		
Education level[n(%)]			0.054	0.815
Junior high school and below	72(68.57)	158(59.62)		
High school and above	33(31.43)	80(30.19)		
Marriage[n(%)]			1.138	0.286
Married	74(70.48)	201(75.85)		
Unmarried/separated/divorced/widowed	31(29.52)	64(24.15)		
Diabetes[n(%)]			2.675	0.102
Yes	38(36.19)	73(27.55)		
No	67(63.81)	193(72.83)		
Hypertension[n(%)]			2.875	0.090
Yes	46(43.81)	123(46.42)		
No	59(56.19)	142(53.58)		
Hyperlipidemia[n(%)]			0.607	0.436
Yes	24(22.86)	51(19.25)		
No	81(77.14)	214(80.75)		
Smoking[n(%)]			1.325	0.250
Yes	34(32.38)	70(26.42)		
No	71(67.62)	195(73.58)		
Drinking[n(%)]			0.006	0.940
Yes	23(21.90)	59(22.26)		
No	82(78.10)	206(77.84)		
Family history of depression			1.599	0.206
Yes	22(20.95)	41(15.47)		
No	83(79.05)	224(84.53)		
VAS score (points)	6.47 ± 1.58	5.87 ± 1.71	3.097	0.002
ODI index	50.33 ± 10.85	45.33 ± 9.46	4.394	<0.001
PSQI score (points)	12.90 ± 2.79	8.75 ± 2.76	13.034	<0.001
Sleep duration [h, n (%)]			35.372	<0.001
≤6	25(23.81)	53(20.00)		
7-8	46(43.81)	188(70.94)		
>8	34(32.38)	24(9.06)		
*Qi* deficiency[n(%)]	47(44.76)	40(15.09)	36.804	<0.001
Blood stasis constitution[n(%)]	44(41.90)	37(13.96)	34.339	<0.001
Phlegm dampness[n(%)]	22(20.95)	41(15.47)	1.599	0.206
Damp-heat constitution[n(%)]	19(18.10)	45(16.98)	0.065	0.798
Other[n(%)]	18(17.14)	41(15.47)	0.157	0.692

BMI, Body Mass Index; VAS, Visual Analog Scale; ODI, Oswestry Disability Index; PSQI, Pittsburgh Sleep Quality Index.

### Statistical methods

Statistical analyses were performed using SPSS version 27.0. Continuous variables with a normal distribution are expressed as mean ± standard deviation, and independent-sample t-tests were applied. Categorical variables are expressed as percentages (%) and analyzed using the χ² test. A total of 370 participants were randomly divided into a training set (n=266) and an internal validation set (n=104) at a 7:3 ratio. An additional 156 participants were collected as a temporal validation cohort to assess model stability over time. All data preprocessing steps (including standardization parameter estimation and feature selection) were performed exclusively in the training set to avoid data leakage. Feature Selection and Model Construction: In the training set, the following steps were used to select features: ① LASSO regression with 10-fold cross-validation was applied to retain features with non-zero coefficients; ② Univariate logistic regression (P<0.05) was performed to identify relevant variables; ③ The features selected from the above steps were jointly entered into multivariable logistic regression (enter method, α=0.05) to determine the final set of risk factors (this step was used only for feature set determination, not as the final classification model). Based on the final set of features, three classification models were constructed: gradient boosting machine (GBM), random forest (RF), and multivariable logistic regression (as a baseline comparator).

Feature importance analysis: The RF model assesses feature importance using the Mean Decrease Gini, with higher values indicating a greater contribution of the feature to the model’s classification. The GBM model evaluates feature importance using relative influence, with higher scores indicating a greater value of the feature in identifying depression. Hyperparameter Tuning: Optimal hyperparameters were determined using 10-fold cross-validation combined with grid search. Tuned parameters for GBM included n.trees (1000), interaction.depth (1–5), and shrinkage (0.01–0.1); tuned parameters for RF included ntree (500–1500) and mtry (1–4). The final optimal parameters were: GBM (n.trees=861, interaction.depth=3, shrinkage=0.05) and RF (ntree=500, mtry=2). All modeling procedures, including feature selection (LASSO regression), hyperparameter tuning (10-fold cross-validation within the training set), and model training, were performed exclusively in the training set (n=266). The internal validation set (n=104) and temporal validation cohort (n=156) were kept completely independent and were not involved in any step of model development. Specifically: ① LASSO feature selection was conducted using only the training set with 10-fold cross-validation; ② hyperparameters for GBM and RF were optimized via grid search based solely on the training set; ③ the final models were locked before any validation set was used; ④ validation sets were used only for final performance evaluation. This strict separation was implemented to prevent data leakage and ensure unbiased performance estimation.

## Results

### Univariate analysis of demographic data and depressive symptoms in middle-aged and older patients with CLBP

In this study, 370 subjects were randomly divided into a training set (n = 266) and validation set (n = 104) at a ratio of 7:3. Among the 370 middle-aged and older CLBP patients, 105 (28.38%) had depressive symptoms, and 265 (71.62%) had did not have depressive symptoms. The VAS score, ODI index, PSQI score, sleep time and the proportion of qi deficiency and blood stasis constitution in the depression group were higher than those in the non-depression group (P < 0.05). The results are presented in **Table 1**.

### Variable screening based on LASSO regression analysis

**Figure 1:**
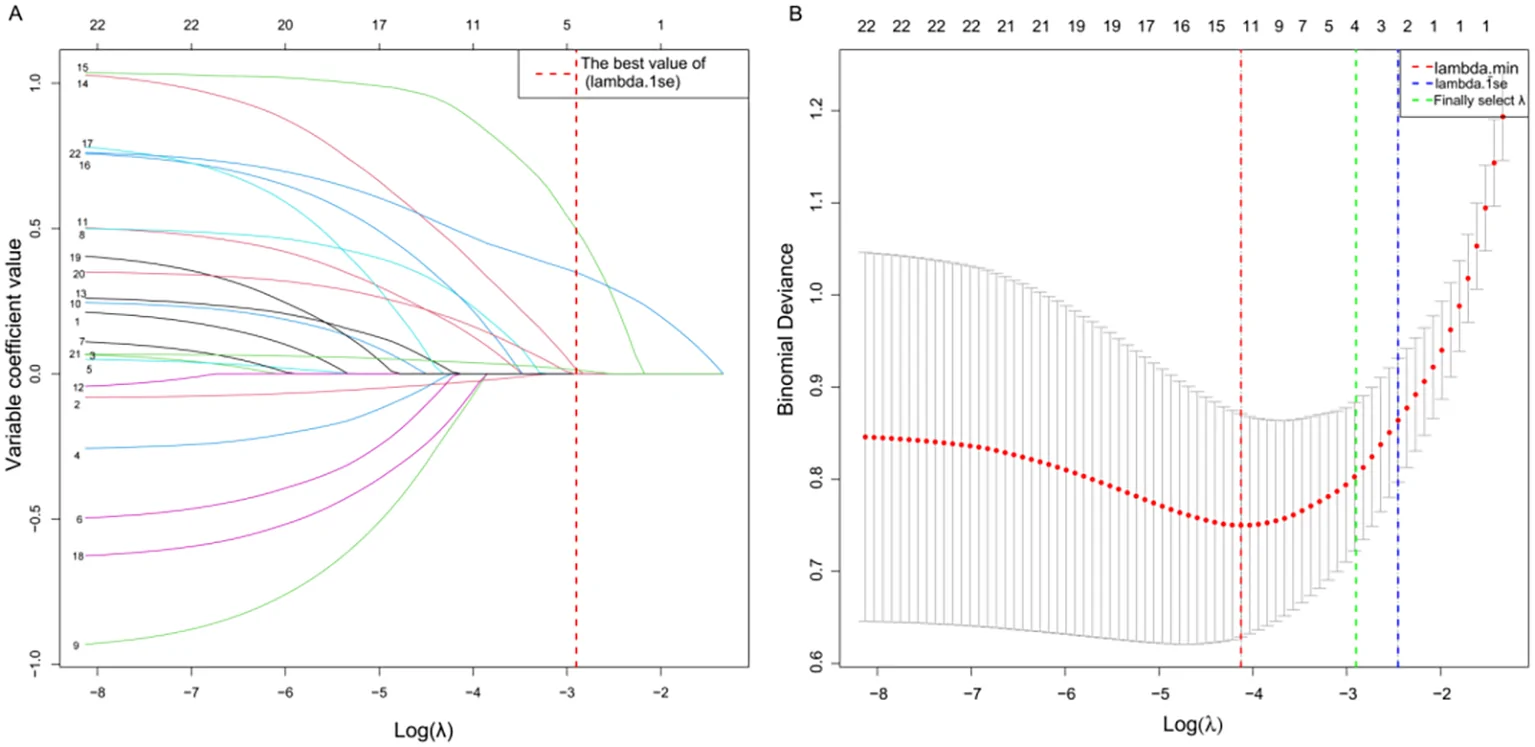
Variable screening based on LASSO regression analysis. **(A)** Dynamic plot of variable selection using LASSO regression. **(B)** Ten-fold cross-validation plot for LASSO regression. ODI, Oswestry disability Index; PSQI, Pittsburgh sleep quality index.

Based on the training set, 22 independent variables in the dataset were included in the LASSO regression analysis to screen out the risk factors influencing depressive symptoms in middle-aged and older patients with chronic low back pain (**Figure 1A**). In the cross-validation curve, the left dotted line represents the optimal λ value of the evaluation index (**Figure 1B**), at which 12 variables had nonzero coefficients. The right dotted line indicates the λ value corresponding to the model within one standard error of the optimal evaluation index, for which the four variables had non-zero coefficients. The four potential variables were the ODI score, PSQI score, qi deficiency, and blood stasis constitution. **Table 2**.

**Table 2:** Variable screening based on LASSO regression analysis.

Factor	λ+1se
ODI Index	0.013
PSQI score	0.349
Qi deficiency constitution	0.017
Blood stasis constitution	0.497

ODI, Oswestry Disability Index; PSQI, Pittsburgh Sleep Quality Index.

### Multivariate logistic regression analysis

Based on the training set, the variables with P < 0.05, as shown in **Table 1**, and those with non-zero coefficients in the LASSO regression in **Table 2** were included in the multivariate logistic regression analysis. The results showed that the VAS score, ODI score, PSQI score, *qi* deficiency, and blood deficiency were independent influencing factors for depression, whereas sleep duration was identified as a protective factor (P < 0.05). The results are presented in **Table 3**.

**Table 3:** Multivariate logistic regression analysis of depressive symptoms in middle-aged and older patients with CLBP.

Factors	β	SE	Wals χ^2^	*P*	OR	95%CI
VAS score	0.257	0.121	4.514	0.034	1.294	1.020~1.648
ODI index	0.056	0.020	7.747	0.005	1.058	1.017~1.100
PSQI score	0.608	0.110	30.470	<0.001	1.837	1.480~2.280
Sleep duration	-1.006	0.499	4.053	0.044	0.366	0.137~0.974
*Qi* deficiency constitution	1.240	0.450	7.578	0.006	3.456	1.429~8.355
Blood stasis constitution	1.422	0.450	9.992	0.002	4.146	1.717~10.014
Constant	-10.558	1.759	30.018	<0.001	0.000	–

VAS, Visual Analog Scale; ODI, Oswestry Disability Index; PSQI, Pittsburgh Sleep Quality Index; CLBP, chronic low back pain.

### Establishment of the GBM model

**Figure 2:**
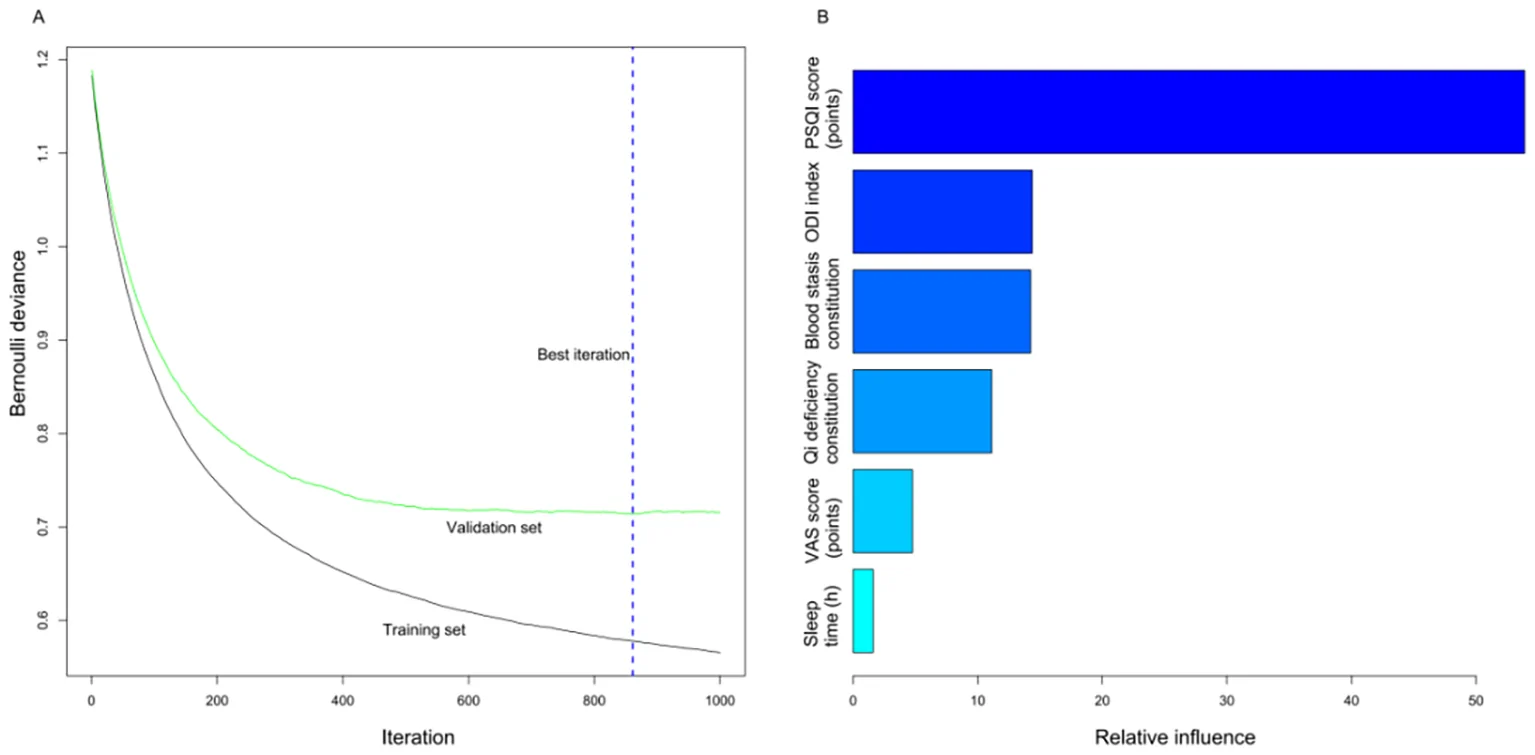
Establishment of the GBM model. **(A)** Dynamic relationship between the classification error of the GBM model and the number of iterations; **(B)** Ranking of variable importance in the GBM model based on relative influence; GBM, gradient boosting machine; VAS, Visual Analog Scale; ODI, Oswestry Disability Index; PSQI, Pittsburgh Sleep Quality Index; CLBP, chronic low back pain.

Based on the 6 risk factors selected by LASSO-Logistic from the training set, a GBM model was established. The shrinkage parameter was set to 0.005 and the initial number of iterations (n.trees) was set to 1000 for model training. A ten-fold cross-validation was applied to determine the optimal number of iterations. When n.trees = 861, the model achieves the smallest classification error, as shown in **Figure 2A**. GBM model: The relative importance of each clinical feature was determined using GBM. In descending order, the feature importance was PSQI score (53.89), ODI score (14.37), blood stasis constitution (14.24), *qi* deficiency constitution (11.13), VAS score (4.76), and sleep duration (1.61), as shown in **Figure 2B**.

### Establishment of the RF model

**Figure 3:**
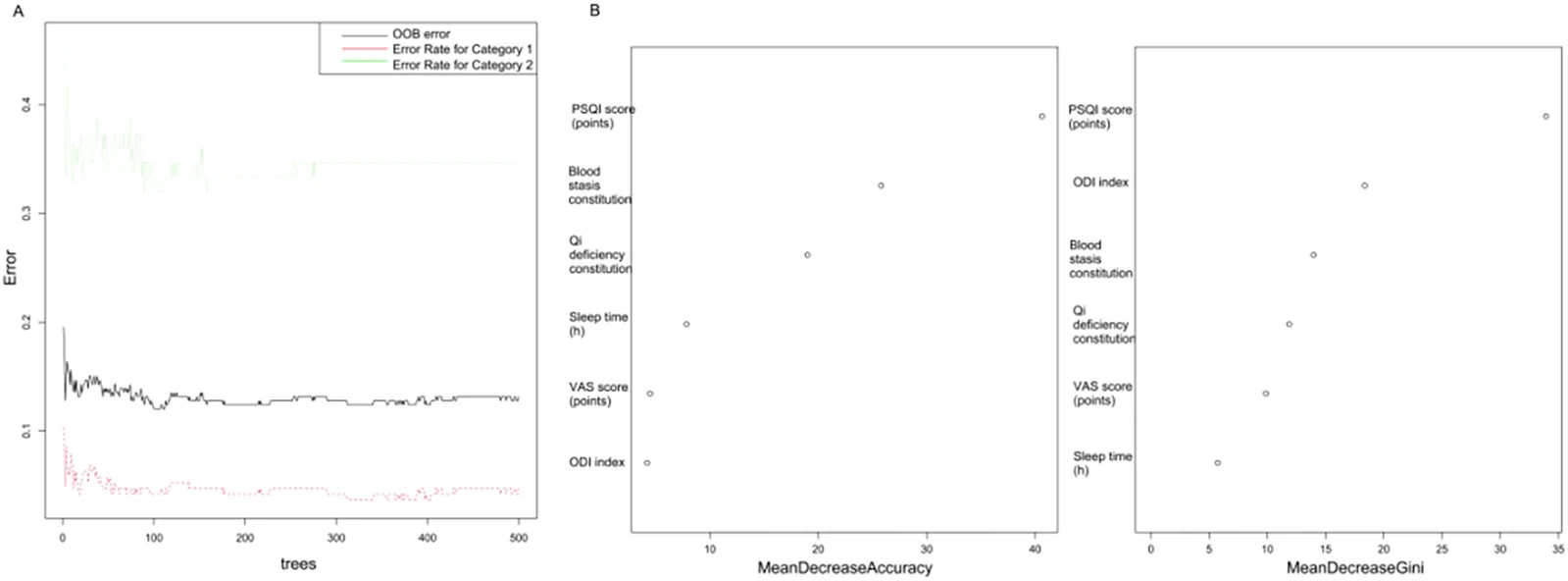
Establishment of the RF model. **(A)** Dynamic relationship between the classification error of the RF model and the number of random trees. **(B)** Ranking of variable importance in the RF model based on the mean decrease in Gini index. RF, random forest; VAS, Visual Analog Scale; ODI, Oswestry Disability Index; PSQI, Pittsburgh Sleep Quality Index; CLBP, chronic low back pain.

Based on the 6 risk factors selected by LASSO-Logistic from the training set, a RF model was established. The number of candidate variables at each node of the trees was set to the square root of the total number of variables and the random seed was set to 12345. When ntree = 500 and mtry = 2, the classification error stabilized, indicating that the model was optimal. The dynamic relationship between the classification error of the random forest and the number of trees is shown in **Figure 3A**.

RF model: The importance of the variables for identifying depressive symptoms was ranked according to the mean decrease in the Gini index. The variables were as follows: PSQI score (26.57), blood stasis constitution (12.77), qi deficiency constitution (10.04), ODI score (8.75), sleep duration (3.90), and VAS score (3.39) (**Figure 3B**).

### Comparison of the classification performance between the GBM and RF models

This study systematically compared the classification performance of logistic regression, GBM, and RF models in the training and validation sets. A summary of all metrics is presented in **Table 4**.

**Table 4:** Comparison of classification performance of three models.

Model	AUC	Accuracy (%)	Sensitivity/recall rate (%)	Specificity (%)	F1	Brier score
Training set						
Logistic regression	0.917(0.875-0.959)	0.872	0.693	0.942	0.754	0.091
GBM	0.927 (0.892-0.961)	0.891	0.693	0.969	0.782	0.087
RF	0.998 (0.997-1.000)	0.951	0.827	1.000	0.905	0.032
Validation set						
Logistic regression	0.850 (0.770-0.930)	0.788	0.533	0.892	0.593	0.139
GBM	0.837 (0.749-0.926)	0.779	0.500	0.892	0.566	0.141
RF	0.811 (0.713-0.909)	0.789	0.433	0.919	0.531	0.150

**Figure 4:**
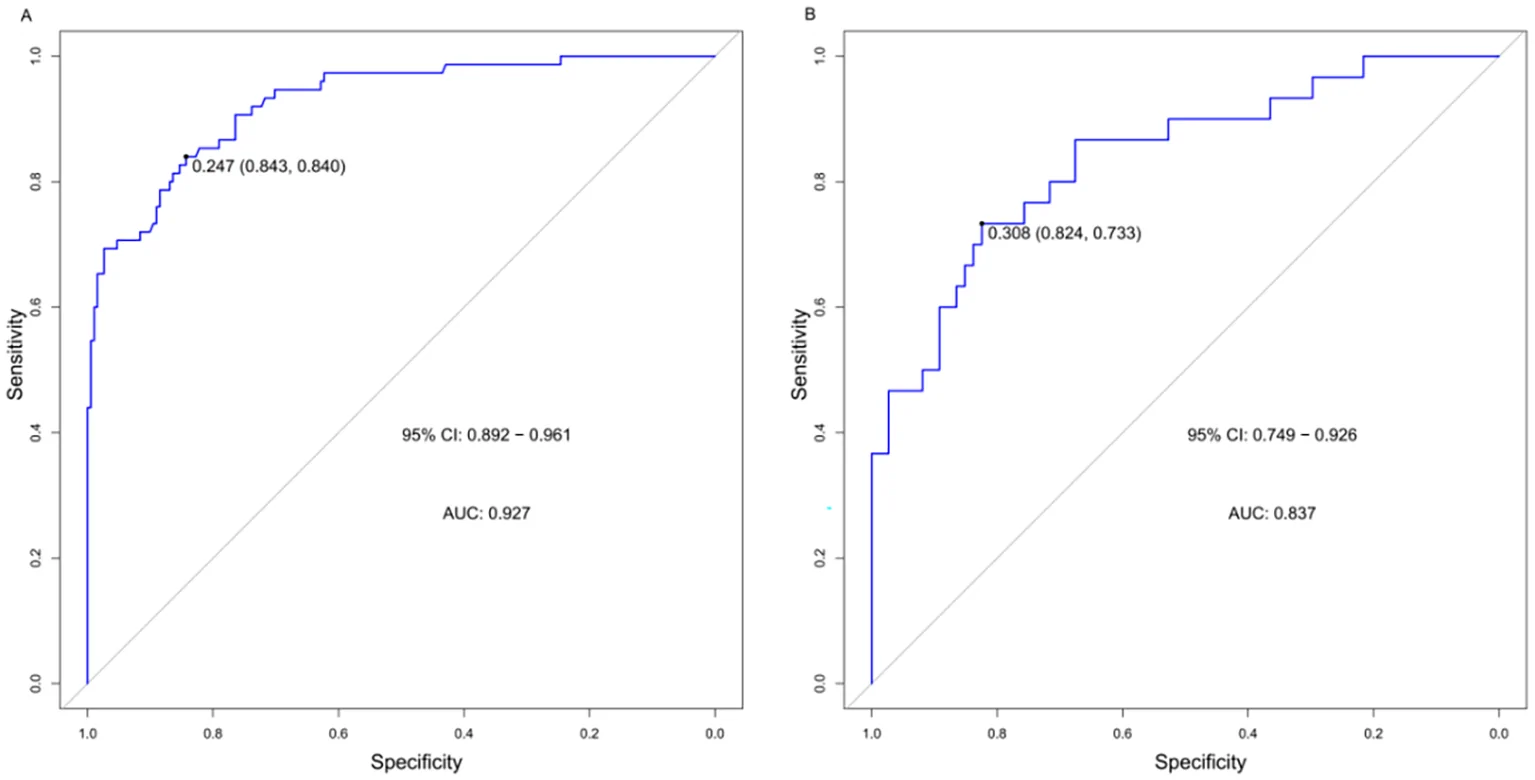
Receiver operating characteristic (ROC) curves of the GBM model for classifying depressive symptoms in middle-aged and older patients with CLBP. **(A)** Training set. **(B)** Validation set.

**Figure 5:**
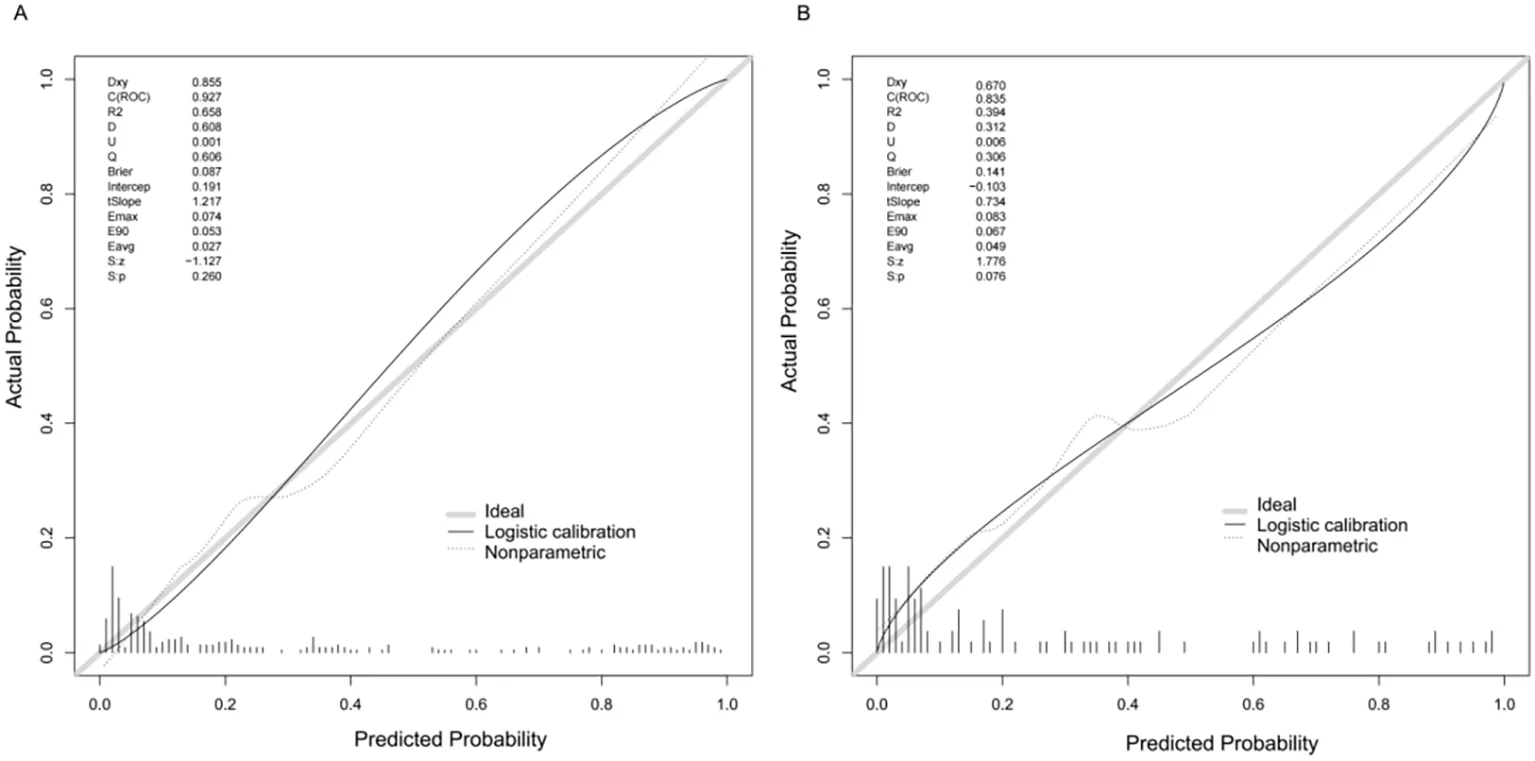
Calibration curves of the GBM model for classifying depressive symptoms in middle-aged and older patients with chronic low back pain. **(A)** Training set. **(B)** Validation set.

The receiver operating characteristic (ROC) curve analysis of the GBM model showed that the area under the curve (AUC) in the training set was 0.927 (95% CI: 0.892–0.961), with a sensitivity of 69.30% and a specificity of 96.90%. In the validation set, the AUC was 0.837 (95% CI: 0.749–0.926), with a sensitivity of 50.00% and a specificity of 89.20%, as shown in **Figure 4**. The calibration curves demonstrated good agreement between the predicted probabilities and observed incidence in both the training and validation sets (**Figure 5**).

**Figure 6:**
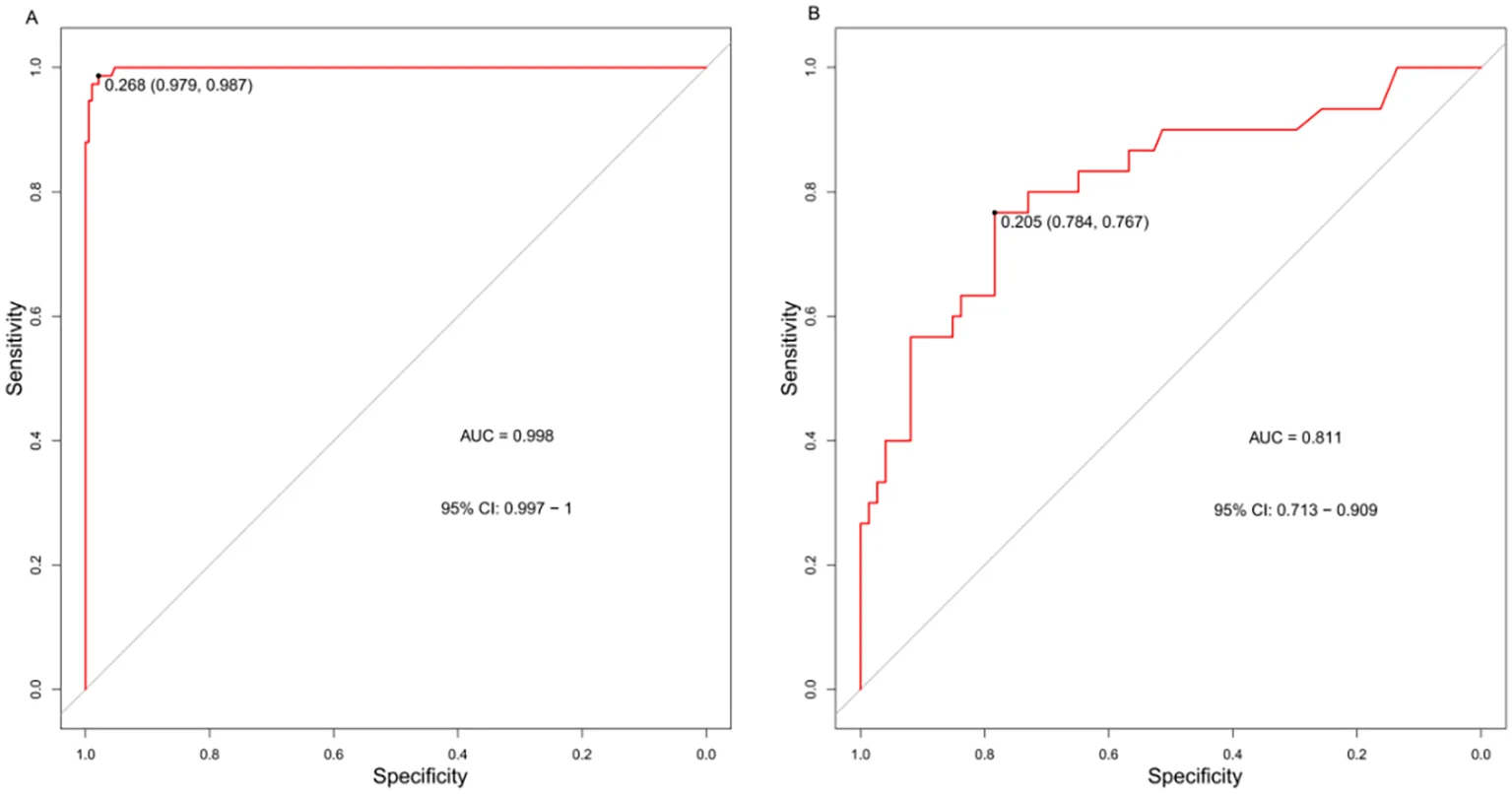
Receiver operating characteristic (ROC) curves of the RF model for classifying depressive symptoms in middle-aged and older patients with CLBP. **(A)** Training set. **(B)** Validation set.

**Figure 7:**
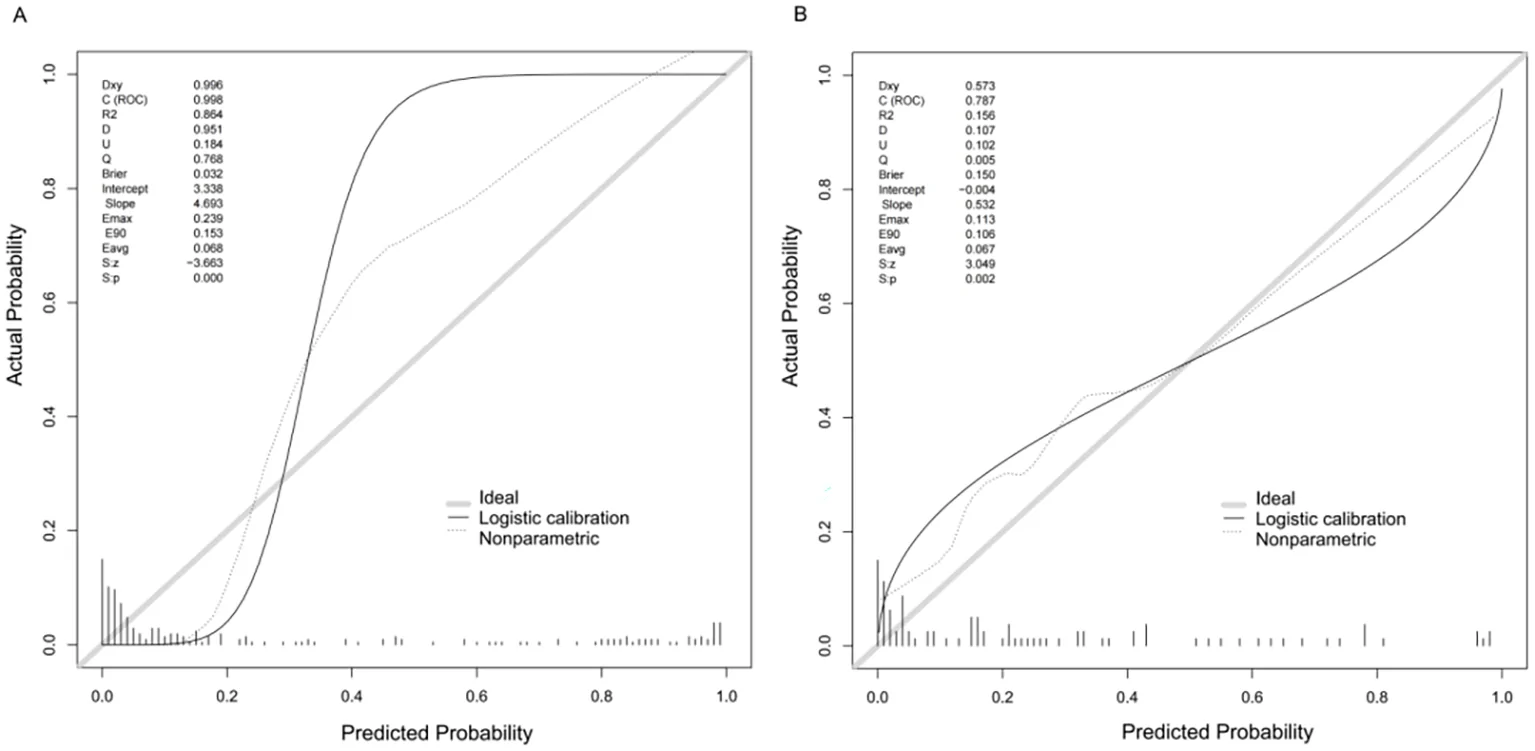
Calibration curves of the RF model for classifying depressive symptoms in middle-aged and older patients with chronic low back pain. **(A)** Training set. **(B)** Validation set.

For the RF model, ROC curve analysis showed that the AUC in the training set was 0.998 (95% CI: 0.997–1.000), with a sensitivity of 82.70% and a specificity of 100.00%. In the validation set, the AUC was 0.811 (95% CI: 0.713–0.909), with a sensitivity of 43.30% and specificity of 91.90%, as shown in **Figure 6**. The calibration curve results indicated good agreement between the predicted probabilities and actual incidence in both the training and validation sets (**Figure 7**).

The logistic regression model, included as a baseline comparator, achieved an AUC of 0.917 in the training set, with an accuracy of 87.20%, sensitivity of 69.30%, and specificity of 94.20%. In the validation set, it achieved an AUC of 0.850, with an accuracy of 77.80%, sensitivity of 53.30%, and specificity of 89.20%.

In the training set, the RF model performed best, with an AUC of 0.998, accuracy of 95.10%, sensitivity of 82.70%, specificity of 100.0%, and F1-score of 0.905. The GBM and logistic regression models showed comparable performance, with AUCs of 0.927 and 0.917, respectively.

In the validation set, the logistic regression model achieved the highest AUC (0.850), with an accuracy of 77.80%, sensitivity of 53.30%, and F1-score of 0.593. The GBM model showed similar performance (AUC 0.837, F1-score 0.566). The RF model performed relatively poorly, with an AUC of 0.811 and a notably low sensitivity of 43.30%, resulting in an F1-score of 0.531.

The Delong test revealed no statistically significant differences in AUC among the three models in the validation set (P > 0.05), indicating comparable classification performance.

The OOB confusion matrix showed that the specificity of the model for the non-depressed group was 96.86%, the sensitivity for the depressed group was 60.00%, and the F1 score was 71.43%. This asymmetric performance suggests that the model tends to make conservative predictions, which may be related to the relatively low proportion of positive samples in the training set (28.20%). The OOB sensitivity (60.00%) was considerably higher than that observed in the internal validation set (43.30%), suggesting a decline in performance when applied to an independent validation sample. This indicates that the RF model may be susceptible to overfitting, and further multi-center external validation is needed to confirm its generalizability.

### Temporal validation

To assess the temporal stability of the model, 156 middle-aged and older patients with CLBP who visited the hospital from July 2024 to June 2025 were additionally collected as the temporal validation cohort. The comparison of baseline characteristics between the original cohort and the time validation cohort is shown in **Table 2**. The results indicated that the standardized mean differences (SMD) of all variables in the two cohorts were all less than 0.20, suggesting that the patient composition was highly comparable. **Table 5**.

**Table 5:** Comparison of baseline characteristics between the original cohort and the temporal validation cohort.

Factors	Time verification (n = 156)	Research cohort (n = 370)	t/χ^2^	P	SMD
Sex[n(%)]			0.013	0.910	0.010
Male	78(50.00)	183(49.46)			
Female	78(50.00)	187(50.54)			
Age (years)	59.09 ± 7.53	58.50 ± 7.28	0.409	0.683	0.080
The course of low back pain [month, n (%)]			0.021	0.884	0.014
>12	66(42.31)	154(41.62)			
≤12	90(57.69)	216(58.38)			
BMI [kg/m2, n(%)]			0.139	0.987	0.022
≤18.4	19(12.18)	43(11.62)			
18.5-23.9	79(50.64)	193(52.16)			
24.0-27.9	36(23.08)	85(22.97)			
≥28	22(56.41)	49(13.24)			
Education level[n(%)]			0.009	0.925	0.009
Junior high school and below	109(69.87)	257(69.46)			
High school and above	47(30.13)	113(30.54)			
Marriage[n(%)]			0.021	0.885	0.014
Married	115(73.72)	275(74.32)			
Unmarried/separated/divorced/widowed	41(26.28)	95(20.27)			
Diabetes[n(%)]			0.170	0.680	0.040
Yes	44(28.21)	111(30.00)			
No	112(71.79)	259(70.00)			
Hypertension[n(%)]			0.029	0.865	0.016
Yes	78(50.00)	182(49.19)			
No	78(50.00)	188(50.81)			
Hyperlipidemia[n(%)]			0.311	0.577	0.051
Yes	35(22.44)	75(20.27)			
No	121(77.56)	295(79.73)			
Smoking[n(%)]			3.146	0.076	0.168
Yes	56(35.90)	104(28.11)			
No	100(64.10)	266(71.89)			
Drinking[n(%)]			0.176	0.675	0.041
Yes	32(20.51)	82(22.16)			
No	124(79.49)	288(77.84)			
Family history of depression			3.056	0.080	0.167
Yes	65(41.67)	185(50.00)			
No	91(58.33)	185(50.00)			
VAS score (points)	5.96 ± 1.60	6.04 ± 1.70	0.520	0.603	0.048
ODI index	46.95 ± 10.80	46.75 ± 10.11	0.200	0.841	0.019
PSQI score (points)	9.99 ± 3.48	9.93 ± 3.34	0.206	0.837	0.018
Sleep duration [h, n (%)]			0.775	0.679	0.079
≤6	38(24.36)	78(21.08)			
7-8	93(59.62)	234(63.24)			
>8	25(16.03)	58(15.68)			
*Qi* deficiency[n(%)]	34(21.79)	87(23.51)	0.183	0.669	0.041
Blood stasis constitution[n(%)]	39(25.00)	81(21.89)	0.602	0.438	0.073
Phlegm dampness[n(%)]	24(15.38)	63(17.03)	0.214	0.643	0.043
Damp-heat constitution[n(%)]	24(15.38)	64(17.30)	0.288	0.591	0.051
Other[n(%)]	27(17.31)	59(15.95)	0.149	0.700	0.038

### Verification result

**Figure 8:**
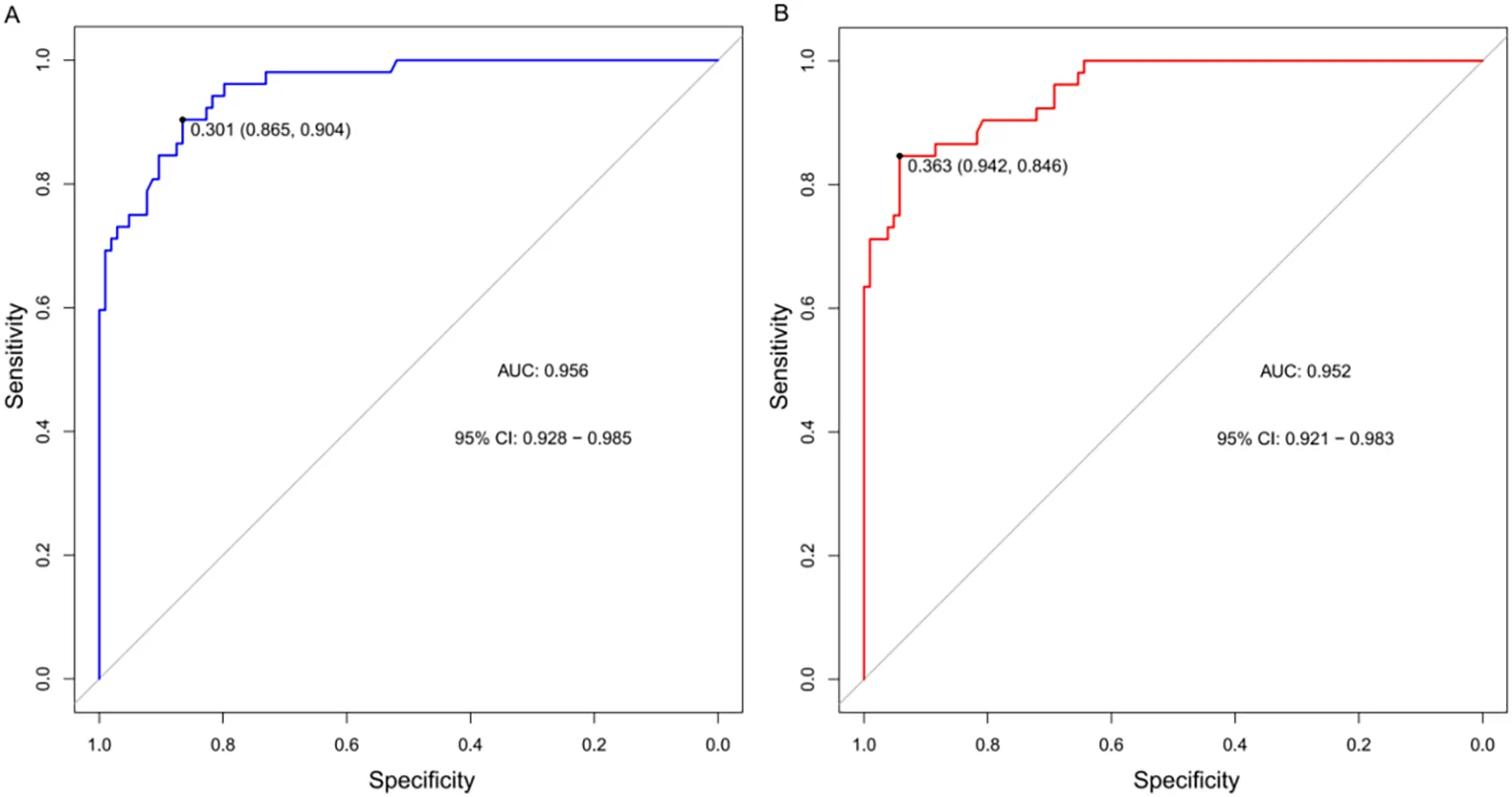
Receiver operating characteristic (ROC) curves of the GBM and RF models for classifying depressive symptoms in middle-aged and older patients with CLBP. **(A)** ROC curve of the GBM model. **(B)** ROC curve of the RF model.

**Figure 9:**
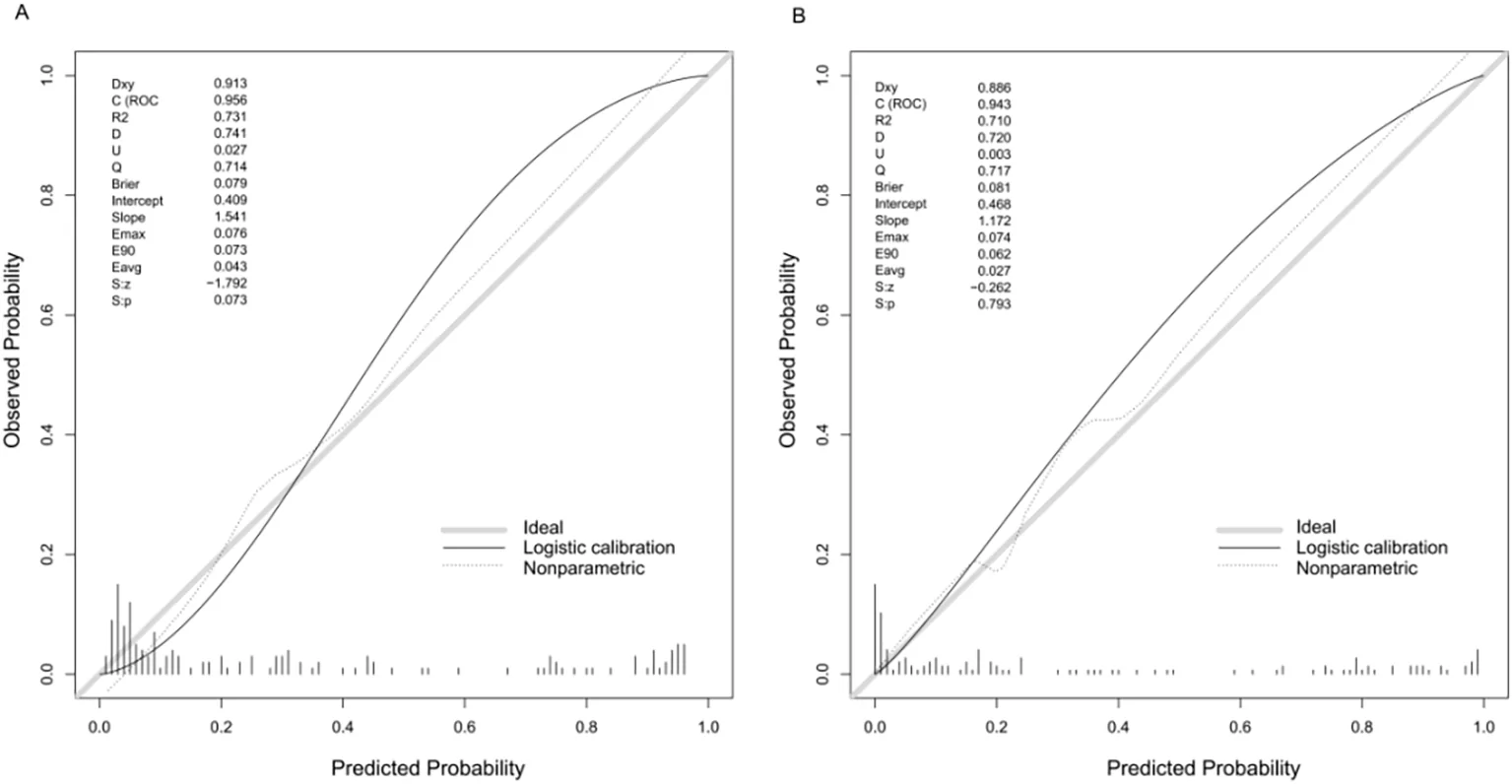
Receiver operating characteristic (ROC) curves of the GBM and RF models for classifying depressive symptoms in middle-aged and older patients with CLBP. **(A)** ROC curve of the GBM model. **(B)** ROC curve of the RF model.

The results of time validation showed that the AUC of the GBM model was 0.956 (95% CI: 0.928–0.985), with a sensitivity of 86.50%, a specificity of 90.40%, and an F1 score of 0.832. The AUC of the RF model was 0.952 (95% CI: 0.921–0.983), with a sensitivity of 94.20%, a specificity of 84.60%, and an F1 score of 0.863. The optimal probability thresholds for calculating sensitivity and specificity were determined in the temporal validation cohort using the Youden index (GBM: 0.301; RF: 0.392). The Brier scores for the GBM and RF models were 0.079 and 0.081, respectively, indicating good calibration. These results indicated that both models demonstrated good discriminative ability (**Figure 8**). The calibration curves showed good agreement between the predicted and observed occurrence probabilities for both models (**Figure 9**). The DeLong test showed no statistically significant difference in AUC between the two models (P = 0.751 > 0.05), indicating comparable classification performance of the GBM and RF models in the temporal validation cohort.

## Discussion

CLBP is a common musculoskeletal disorder in middle-aged and older populations. The global prevalence of CLBP is 38.9%, which increases with age (14). Patients with CLBP not only experience limitations in physical function but also frequently develop depressive symptoms due to long-term pain stimulation, sleep disturbances, and psychological stress, which seriously affect their physical and mental health (15). The presence of depressive symptoms can form a vicious cycle of “pain–emotional disorder–delayed rehabilitation,” leading to reduced treatment compliance, increased medical costs, and even a higher risk of adverse events such as falls and suicide (16). Currently, the clinical identification of depressive symptoms in middle-aged and older patients with CLBP relies mainly on subjective scale assessments that lack objective and efficient early screening tools. Moreover, existing studies often focus on single-dimensional indicators, neglecting the complementary value of multimodal characteristics in Chinese and Western medicine (17, 18). Therefore, this exploratory study integrates multidimensional features of TCM syndromes, Western medicine clinical indicators, and physical function, uses LASSO regression to screen key variables, and constructs two machine learning classification models, GBM and RF, to provide a preliminary screening tool for screening depressive symptoms in middle-aged and older patients with CLBP.

In this study, LASSO regression and multivariate logistic regression analyses showed that VAS score, ODI score, PSQI score, *qi* deficiency, and blood stasis were independent risk factors for depressive symptoms in middle-aged and older patients with CLBP, while sleep duration was a protective factor (P < 0.05). From the perspective of Western medicine, higher VAS scores indicate greater pain intensity. Persistent severe pain can activate the hypothalamic–pituitary–adrenal (HPA) axis, leading to increased serum cortisol levels, thereby inhibiting the synthesis and release of neurotransmitters such as 5-hydroxytryptamine and dopamine and inducing depression (19). An increased ODI score reflects a significant decline in daily living ability and is a core indicator for evaluating physical dysfunction in patients with CLBP. Loss of self-care capacity, social isolation, and related problems can further aggravate feelings of loneliness and helplessness, increasing the risk of depression (20). An elevated PSQI score indicated poor sleep quality. There is a bidirectional vicious cycle between pain and sleep disorders: pain leads to difficulty falling asleep and sleep fragmentation, whereas sleep deprivation lowers the pain threshold and amplifies negative emotional perception, ultimately inducing depression (21). In addition, adequate sleep duration as a protective factor can promote neurotransmitter balance, improve emotional regulation, and reduce the risk of depression. This finding is consistent with previous studies showing that “sleep duration ≥7 h/night can reduce the incidence of depression in patients with chronic pain” (22, 23). Recent machine learning studies have also explored depression classification in chronic pain populations. For instance, Goldstein et al. applied machine learning to screen future pain in chronic back pain patients, reporting screening accuracies of 65-72% (24). These findings support the potential value of machine learning approaches in this clinical context.

From the perspective of TCM theory, patients with a *qi* deficiency constitution, characterized by “insufficiency of vital energy and decline in the functions of internal organs,” often present with fatigue, shortness of breath, and reluctance to speak. Such individuals generally have lower tolerance to pain. When exposed to prolonged CLBP stimulation, the deficiency of vital energy is insufficient to resist “emotional pathogenic factors,” making these patients prone to emotional abnormalities such as “weak will be due to *qi* deficiency” (25). Patients with a blood stasis constitution, due to qi stagnation and blood stasis obstructing the meridians, are prone to insomnia, frequent dreaming, and low mood, which is analogous to the Western medicine mechanism of “insufficient neurotransmitter synthesis” (26). Notably, blood stasis constitution ranked third in the variable importance analysis. Although it was not identified as an independent risk factor in the multivariate logistic regression, it still suggested an association with depressive symptoms. This may be related to the concept of “long-term pain entering the meridians, leading to blood stasis and *qi* stagnation.” CLBP can cause impaired circulation of *qi* and blood, and blood stasis may obstruct *qi* movement, resulting in abnormal emotional expression and subsequent inducing of depression (27). These findings provide a theoretical basis for TCM constitution-based intervention. These findings provide preliminary theoretical support for the potential relevance of TCM constitution-based approaches, though further prospective studies are needed to confirm their clinical utility.

In recent years, machine learning methods have been increasingly applied in the prediction of depression in patients with chronic pain (9, 10, 28). However, the aforementioned studies have significant differences in population characteristics, research design, and time dimension compared to this study. Their results should be regarded as a background reference rather than a direct validation of this model. Additionally, pain intensity, sleep quality, and functional impairment have been proven to be important risk factors for depression in patients with chronic pain, but their effect sizes may vary among different pain subtypes (29), which further supports the necessity of conducting targeted modeling in the specific population of CLBP. In this context, the constructed model should be positioned as a preliminary exploratory tool for cross-sectional screening of depressive symptoms in middle-aged and elderly patients with CLBP.

Both the GBM and RF models demonstrated promising preliminary classification performance. The GBM model achieved AUC values of 0.927 and 0.837 in the training and validation sets, respectively, while the RF model achieved 0.998 and 0.811. Delong test revealed no statistically significant differences in predictive performance between the two models in the validation set (P > 0.05), indicating comparable classification efficacy for screening depressive symptoms in middle-aged and elderly CLBP patients.

Although only six predictors were retained after LASSO selection, we prioritized GBM and RF as the primary classification models for the following reasons: ① Clinical factors may exhibit complex nonlinear relationships with depressive symptoms, which machine learning models can better capture; ② Ensemble methods are particularly effective at modeling interactions among features; ③ We also report logistic regression model performance as a baseline comparator.

Furthermore, time validation demonstrated that the GBM and RF models achieved similar AUC values (0.956 and 0.952, respectively), with no statistically was significant difference (P = 0.751), indicating that both models have comparable classification performance in the temporal cohort. This finding differs from our original observation, and the corrected results suggest that the two algorithms perform similarly in this clinical context. The OOB evaluation of the RF model showed an error rate of 13.53% and an accuracy of 86.47%, with a sensitivity of 60.00% and a specificity of 96.86%, suggesting that the overfitting risk of the model is acceptable, though moderate performance decline was observed when applied to the independent validation set. The comparable performance between GBM and RF may be attributed to their distinct yet complementary working principles. The RF adopts a bagging ensemble strategy, enhancing model robustness and resistance to interference through the construction and voting of multiple decision trees (30). In contrast, GBM uses a sequential boosting strategy that improves the fitting accuracy but is more sensitive to data noise (31, 32). Under the condition of limited temporal validation samples in this study, GBM may be affected more by data specificity, resulting in greater performance fluctuations. Conversely, the autonomous sampling mechanism of the RF confers greater stability in small-sample validation. In addition, the inherent anti-overfitting advantage of RF when handling complex interactions among clinical variables also contributed to its superior performance. However, the relatively low sensitivity of the RF model in the validation set (43.30%) indicates that nearly half of the depressed patients would be missed if the model were applied in clinical practice, which is a notable limitation. This may be partially attributed to the imbalanced outcome distribution in the training set (28.20% positive), which predisposes the model to favor the majority class. Therefore, the current model is best suited for preliminary screening rather than diagnostic confirmation, and further optimization of the decision threshold or the use of cost-sensitive learning approaches may be considered in future studies to improve sensitivity.

However, this study has several limitations. First, as an exploratory cross-sectional study, the findings should be interpreted as hypothesis-generating rather than confirmatory. The model has not been prospectively validated in real-world clinical settings and should not be interpreted as a decision-making tool for clinical intervention at this stage. ① Single-center cross-sectional design: The study was conducted at a single center with a relatively limited sample size, which may introduce selection bias. The cross-sectional design precludes causal inference. The temporal validation cohort was derived from the same institution and represented only a temporal split of the data, which cannot be considered truly independent external validation. The high homogeneity between the original and temporal validation cohorts (all SMDs < 0.20) may partially explain the high AUC observed in temporal validation and further highlights the need for geographically and institutionally independent external validation. Future multi-center, prospective cohort studies are needed to further validate model generalizability. ② Sample size considerations: No formal sample size calculation was performed *a priori*; the sample size was determined by the available data. However, the training set contained 75 outcome events with 6 candidate predictors (EPV = 12.5), which exceeded the recommended minimum of 10 events per variable. ③ Outcome measurement error: The outcome was defined based on a PHQ-9 screening threshold (≥10) rather than a specialist-confirmed clinical diagnosis using structured interviews, which may introduce measurement bias and misclassification. Future studies should consider using gold-standard diagnostic methods for validation. ④ Subjectivity in TCM feature assessment: TCM constitution and syndrome classification relied on physician assessment. Although standardized scales and consistency checks were used, objective quantitative indicators (e.g., digital tongue and pulse data) are lacking. Future studies could incorporate TCM diagnostic instruments to improve objectivity. ⑤ Omission of psychosocial factors: The model did not include psychosocial factors such as social support, economic status, or family caregiving level, which may influence depressive symptoms through psychological adjustment. Future studies could incorporate these variables. ⑥ Interpretation of high AUC values: Although measures such as strict data separation and 10-fold cross-validation were implemented to rule out information leakage and overfitting, and the high AUC is partially supported by strong associations between core variables and depressive symptoms, multi-center studies with larger sample sizes are needed to further validate model stability. ⑦Feature importance interpretation: The feature importance rankings derived from GBM and RF models differed, which is consistent with their distinct algorithmic mechanisms (bagging vs. boosting). Therefore, the results of feature importance should be interpreted with caution and primarily used for understanding model behavior rather than for causal inference. ⑧ Lack of clinical utility validation: Clinical utility validation was not conducted. The model could be considered for future integration into clinical workflows as a risk stratification tool, with model outputs serving as clinical references rather than diagnostic determinants. However, these applications remain speculative at this stage. Future randomized controlled trials are needed to evaluate the effectiveness of model-based intervention strategies (e.g., targeted pain management, sleep interventions, constitution-based therapies) in reducing depression incidence.

## Conclusion

The VAS, ODI, PSQI, qi deficiency constitution, and blood stasis constitution were identified as independent risk factors for depressive symptoms in middle-aged and older patients with chronic low back pain, whereas sleep duration served as a protective factor. The integration of multimodal characteristics using GBM and RF modeling approaches demonstrated promising preliminary classification performance, suggesting their potential utility in assisting clinicians in identifying high-risk populations and informing clinical decision-making. Further multi-center prospective studies are needed to confirm the generalizability of these findings.

## Data Availability

The raw data supporting the conclusions of this article will be made available by the authors, without undue reservation.
